# Metagenomic profiling of ocular surface microbiome changes in *Demodex* blepharitis patients

**DOI:** 10.3389/fcimb.2022.922753

**Published:** 2022-07-22

**Authors:** Yana Fu, Jie Wu, Dandan Wang, Tiankun Li, Xinwei Shi, Lu Li, Minying Zhu, Zuhui Zhang, Xinxin Yu, Qi Dai

**Affiliations:** ^1^ School of Optometry and Ophthalmology, the Eye Hospital of Wenzhou Medical University, Wenzhou, China; ^2^ Eye Center, The Second Affiliated Hospital, Zhejiang University School of Medicine, Hangzhou, China; ^3^ Ophthalmology Department, The First Affiliated Hospital of Soochow University, Suzhou, China; ^4^ Tianjin Eye Hospital, Tianjin Key Lab of Ophthalmology and Visual Science, Tianjin Eye Institute, Nankai University Affiliated Eye Hospital, Tianjin, China; ^5^ College of Mathematical Medicine, Zhejiang Normal University, Jinhua, China

**Keywords:** *Demodex* blepharitis, meibum, metagenomic next-generation sequencing, microbial communities, ocular surface

## Abstract

**Purpose:**

To compare the ocular surface and meibum microbial communities of humans with *Demodex* Blepharitis (DB) and healthy controls.

**Methods:**

Conjunctival sac and meibum samples from 25 DB patients and 11 healthy controls were analyzed using metagenomic next-generation sequencing (mNGS).

**Results:**

The alpha-diversity of the conjunctival sac microbiome of the *DB* group (observed, Chao1, ACE) was lower than that of the control group, whereas all meibum diversity indicators were similar. In conjunctival samples, the relative abundance (RA) of the phylum Proteobacteria was significantly higher (*p*=0.023), and the RA of both phyla Actinobacteria and Firmicutes was significantly lower (*p*=0.002, 0.025, respectively) in the DB group than that in the control group. In meibum samples, the RA of the phyla Proteobacteria and Actinobacteria were similar, whereas that of the phylum Firmicutes was significantly lower in the DB group (*p*=0.019) than that in the control group. Linear discriminant analysis with effect size measurement of the conjunctival and meibum microbiomes showed that *Sphingobium* sp. *YG1* and *Acinetobacter guillouiae* were enriched in the DB group. *Sphingobium* sp. *YG1, Acinetobacter guillouiae* and *Pseudomonas putida* in the DB group were related to more severe ocular surface clinical parameters. Discriminative genera’s principal coordinate analysis separated all control and DB microbiomes into two distinct clusters.

**Conclusions:**

Proteobacteria’s increased prevalence may indicate ocular microbial community instability. The species *Sphingobium* sp. *YG1* and *Acinetobacter guillouiae* are potentially pathogenic bacterial biomarkers in DB. *Demodex* infection mainly affects the ocular surface microbiome rather than penetrating deeper into the meibomian gland.

## Introduction

The ocular surface microbiome is an important component of the ocular surface. In healthy people, microbes coexisting in the ocular surface or other organs maintain a stable state of competition and cooperation, such as competition with each other or mutual use of resources, nutrition, and space (Lee et al., 2012; Li et al., 2019). The relative abundance (RA) of each member of the microbiome and interactions among microbial species are crucial for the homeostasis and sustainability of ecosystems (Wintermute and Silver, 2010; Kumar et al., 2016). Disruption of the stable state can lead to ocular surface diseases, such as dry eye, meibomian gland dysfunction (MGD), and blepharitis (Zhu et al., 2018; de Paula et al., 2019; Yan et al., 2020).

Blepharitis is a chronic inflammatory process of the eyelid margin that may result in tear film changes, eye irritation symptoms, clinically apparent inflammation, and ocular surface disease (Amescua et al., 2019). An important etiological factor of blepharitis is *Demodex* mites (Coston, 1967; Rodríguez et al., 2005; Zhao et al., 2012). *D. folliculorum* and *D. brevis* are the most common ectoparasites on the ocular surface (Cheng et al., 2015). *D. folliculorum* primarily inhabits lash follicles, whereas *D. brevis* infests lash sebaceous and meibomian glands (MGs). The probable pathogenic role of *Demodex* in blepharitis includes direct mechanical abrasion by mite claws and MG orifice obstructions, inflammatory responses elicited by mite debris or waste, and bacteria carried by *Demodex mites* (Zhu et al., 2018). Therefore, the identification of microbial communities on the ocular surface of *Demodex* blepharitis (DB) patients can help to clarify the pathological mechanisms and provide valuable information for prevention or treatment.

Culture-based and culture-independent approaches, including polymerase chain reaction, 16S rRNA sequencing, and denaturing gradient gel electrophoresis, have been applied to study ocular microbial communities (Zhang et al., 2017; Jiang et al., 2018; Zhu et al., 2018; Kang et al., 2021). Development of culture-independent approaches, such as 16S rRNA comparison, has demonstrated that the ocular surface microbiota is more diverse in composition than as deduced from culture-based methods (Yan et al., 2020; Kang et al., 2021). Nowadays, a few studies had investigated the effect of *Demodex* mites on ocular surface flora by 16S rRNA or culture methods, but the results were quite different (Zhu et al., 2018; Yan et al., 2020; Liang et al., 2021). Compared to 16s rRNA sequencing, metagenomic next-generation sequencing (mNGS) has the advantage of sequencing all genomic DNA in a given sample, leading to a higher resolution and detection of more species, and also including taxa of the viruses, eukaryotes, and superkingdoms archea (Ranjan et al., 2016). To our best knowledge, mNGS had never been used to investigate the microbial communities in the ocular surface and meibum of DB patients. Here, we applied mNGS technology to compare the diversity and interactions of microbial communities in conjunctival swabs and meibum obtained from DB patients and healthy controls.

## Methods

### Sample collection

Twenty-five DB patients who visited the Eye Hospital, Wenzhou Medical University for ophthalmic examinations between June 2019 and May 2020 and 11 healthy controls without blepharitis or MGD were enrolled in our study. Informed consent was obtained from all the participants. This study was approved by the Ethical Committee of the Eye Hospital of Wenzhou Medical University and registered at ClinicalTrials.gov: NCT04451122. All methods were conducted in accordance with the tenets of the Declaration of Helsinki. *Demodex* blepharitis was diagnosed based on the diagnostic criteria (presence of at least one symptom, such as redness, eye itching, foreign body sensation, abnormal eyelashes with cylindrical dandruff in both eyes, and positive results on light microscopic examination of the eyelashes) and age >20 years (Liang et al., 2017). According to the diagnostic criteria, three lashes with retained cylindrical dandruff were removed from each lid and placed separately on each end of a glass slide for a total of 12 lashes on four slides. If at least three or more *Demodex* bodies, including adult, larva, protonymph, or nymph stage of *D. folliculorum* or *D. brevis*, were found on any of the four slides, the result was considered positive. Control participants had never been diagnosed with blepharitis or MGD, were aged >20 years, and had clean eyelashes without cylindrical dandruff or squamous cell debris (collarette). Participants wearing contact lenses, with chalazion, active ocular or nasolacrimal infections, severe systemic diseases, a history of probiotic treatment within the previous 6 months, systemic antibiotic drugs within the previous 3 months, or topical antibiotics within the previous week were excluded from the study. Participants were asked to withhold any topical medications for 48 h before sample collection. To avoid contamination during sample collection, the samples were collected in an ophthalmic treatment room sterilized with ultraviolet light twice a day for at least one hour each time, and for more than half an hour before each sampling. Conjunctival swab samples were collected using sterile transport swabs (IngeniGen XMK Biotechnologies Inc. Zhejiang, China) and were taken from the lower conjunctival sac and MGs. MG secretions from the first squeeze were discarded to avoid contamination from eyelid margins. Another sterile swab was used to collect the secretions from the second squeeze, which was thought to be the meibum in the deep segment of MGs The sterile swabs containing the samples were immediately placed into a sterile tube containing preservation solution and were stored in an ultralow temperature freezer at -80°C before DNA extraction.

### Participant examination

Clinical assessments were performed sequentially as follows: SPEED questionnaire, fluorescein tear break-up time (TBUT), corneal fluorescein staining (CFS) (Song et al., 2013), lid abnormality (LAM) (Fu et al., 2021), meibum expressibility, and meibography. We assessed the meibum expressibility of 15 glands in each lower eyelid (0–45) (Lane et al., 2012). Images of upper and lower MGs were captured using Keratograph 5M (K5M; Oculus, Wetzlar, Germany). The meiboscore was determined as follows: 0, no MG atrophy; 1, MG atrophy of <1/3 of the total lid area; 2, MG atrophy of 1/3 to 2/3 of the total lid area; 3, MG atrophy of > 2/3 of the total lid area. The upper and lower eyelids’ scores were added to yield the final meiboscore (range, 0–6) (Arita et al., 2008).

### DNA extraction and mNGS Analysis

DNA was extracted from the swabs using a DNA extraction kit (IngeniGen XMK Biotechnologies Inc., Zhejiang, China), according to the manufacturer’s instructions. Before DNA extraction, an internal control bacterium was added to the samples. DNA concentration was measured using a Qubit^®^ 4.0 Fluorometer (Thermo Fisher Scientific, Waltham, MA). DNA libraries were constructed using the Ingenigen XMKbio DNA-seq Library Prep Kit (IngeniGen XMK Biotechnologies, Inc.) using the Tn5 transposase method. DNA library concentrations were measured using a Qubit^®^ 4.0 Fluorometer (Thermo Fisher Scientific), and their quality was evaluated using an Agilent 4200 TapeStation system (Agilent Technologies, Santa Clara, CA). Qualified libraries with different barcodes were then pooled accordingly. Blank tubes with unused swabs and sterile water were used as blank extraction-negative controls during DNA extraction and library preparation to filter reagent and laboratory environmental contamination taxa. The “environmental” species with a frequency of more than 10% in the negative controls (pre-determined by ingeniSeq-MG V1.0 mNGS software) over the past 100 runs were considered as contaminants and filtered out from the final results. Sequencing was performed on an Illumina Nextseq550 using a 75-bp single-end sequencing mode.

### Bioinformatics and statistical analysis of shotgun metagenomic data

Raw metagenomic shotgun reads were quality-checked and trimmed using fastp (Chen et al., 2018). Sequences were aligned with the human reference genome (GRCh38) using bowtie2 (Langmead and Salzberg, 2012) to remove human genome sequences; thus, the unaligned reads were recovered. Background sequences from run processing were also filtered from the recovered unaligned reads. All non-host reads were assumed to be microbe-related. Kraken2 was used for taxonomic classification (Wood et al., 2019). HUMAnN3 (Beghini et al., 2021) was used for KEGG pathway analysis. Permutational multivariate analysis of variance (PERMANOVA) between HC and DB groups was done with adonis in vegan with a similarity index using 9999 permutations. All further bioinformatics analyses, data visualization, and statistical analyses were performed in R (version 4.0) (Team R Development Core, 2018) using the vegan (Dixon, 2003), ggplot2, and microeco (Liu et al., 2021) packages.

The contaminant filtering step removed samples with insufficient sequences (<10,000) as well as operational taxonomic units present at <0.0001% RA. Data were statistically analyzed using SPSS software (version 20.0; IBM SPSS Corp., Chicago, IL). Independent-sample *t*-tests, chi-square tests, and Mann–Whitney U tests were used to compare the differences in age, sex, and clinical examination results between DB patients and controls. The Mann–Whitney U test was performed to analyze the alpha-diversity indices and relative abundances of dominant phyla, genera, and species between groups. The generalized estimating equation was used to assess the relationship between ocular surface parameters and relative abundances of top species. Statistical significance was set at *p*<0.05.

## Results

### Demographics and participants’ clinical features

Fifty eyes from 25 DB patients and 22 eyes from 11 healthy adults were enrolled in the study. The clinical parameters of DB patients and healthy controls are shown in **Table 1**. The SPEED scores, TBUT, CFS, LAM, meiboscores, and meibum expressibility were significantly higher in DB patients than in controls (**Table 1**).

**Table 1:** Clinical parameters of the two groups in the study population.

Parameters	*Demodex* blepharitis(n = 25)	Control(n = 11)	t/X^2^/Z	*p-*value
Age (years, mean ± SD)	44.909 ± 12.486	28.000 ± 5.604	-5.363	<0.001^*^
Sex (n, male/female)	12/13	5/6	0.020	0.888^†^
SPEED (0–24)	10.04 ± 4.593	4.182 ± 3.970	-3.608	0.001^*^
TBUT (seconds)	3 (2.000, 4.500)	5 (3.583, 8.500)	-3.116	0.002^‡^
CFS (0–12)	0 (0.000, 2.000)	0 (0.000, 0.5000)	-0.734	0.463^‡^
LAM (0–5)	3 (2.000, 3.000)	0 (0.000, 0.000)	-4.758	<0.001^‡^
Meibum expressibility (0–45)	5 (3.000, 16.000)	41.5 (36.500, 45.000)	-4.274	<0.001^‡^
Meiboscore (0–6)	3 (2.000, 4.000)	1 (0.000, 2.000)	-3.732	<0.001^‡^

SPEED, standard patient evaluation of eye dryness; TBUT, tear break-up time; CFS, corneal fluorescein staining; LAM, lid abnormality. ^*^p-values adjusted for by analysis of independent-sample t-tests. ^†^p-values adjusted for by analysis of the chi-square test. ^‡^p-values adjusted for by analysis of the Mann–Whitney U test.

### Taxonomic assignment

Whole metagenomes were generated from the conjunctival swab and meibum of control (n=11) and DB (n=25) patients in this study. Overall, 1249.17 million reads were generated for the 72 samples, with an average of 19.8 million reads per sample. The average RAs of bacteria, fungi, viruses, and unclassified bacteria were 94.9%, 1.0%, 3.8%, and 0.18%, respectively, in conjunctival swabs and 95.5%, 0.8%, 3.2%, and 0.36%, respectively, in meibum groups. Bacterial taxonomic assignment and hierarchical classification of the reads revealed 12 phyla, 222 genera, and 575 species in conjunctival swabs and 11 phyla, 221 genera, and 508 species in meibum in both groups.

### Alpha-diversity

**Figure 1:**
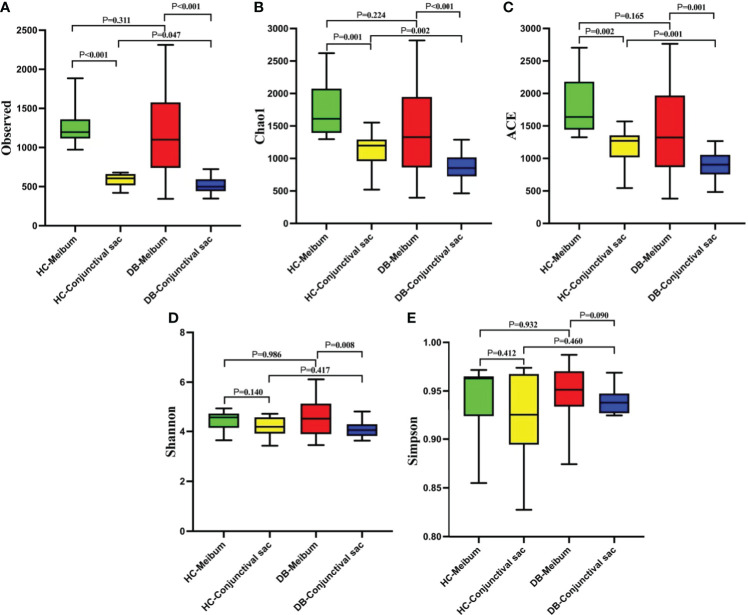
The comparisons of the alpha-diversity indices (A, Observed ; B, Chao1 ; C, ACE ; D, Shannon ; E, Simpson) in the conjunctival sac and meibum microbiome between the two groups. HC, Healthy control; DB, *Demodex* blepharitis.

**Figure 1** shows the comparisons of alpha-diversity of conjunctival swab and meibum samples between the two groups. The conjunctival sac microbiome community population of the DB group (observed, Chao1, ACE) was lower than that of the control group, whereas their community diversities (Shannon and Simpson) were similar (**Figure 1**). In the meibum samples, there was no significant difference in the alpha-diversity between the two groups. The conjunctival sac had lower diversity (observed, Chao1, ACE, Shannon) than the meibum in both groups. Rarefaction curves were plotted for all bacterial microbiomes, and most samples showed a tendency towards saturation, indicating that sufficient depth and coverage had been achieved during sampling.

### Taxonomic composition and linear discriminant analysis effect size analysis of the conjunctival swab or meibum bacterial microbiota

**Table 2** shows that there were five bacterial flora phyla (Proteobacteria, Actinobacteria, Firmicutes, Bacteroidetes, and Deinococcus-Thermus), one phylum from the eukaryotic microbiome (Apicomplexa), and one virus phylum (Uroviricota), which had a RA>1%, in each group. Proteobacteria, Actinobacteria, and Firmicutes were predominant. The differences in the mean abundances of Proteobacteria, Actinobacteria, and Firmicutes between the DB and control groups’ conjunctival swab samples were significant (p<0.05, **Table 2**). Similarly, the difference in the mean abundance of Firmicutes between the DB and control groups’ meibum samples was significant (p<0.05, **Table 2**); however, those of Proteobacteria and Actinobacteria were not significant (**Table 2**).

**Table 2:** Phyla in the conjunctival swab or meibum samples of controls and *Demodex* blepharitis groups.

Taxonomy	RA			*p*-value	RA		*p*-value
	HC-C	DB-C		HC-C vs DB-C	HC-M	DB-M	HC-M vs DB-M
Proteobacteria	65.13	75.35		0.023	69.53	76.02	0.250
Actinobacteria	16.89	6.07		0.002	15.92	10.00	0.050
Firmicutes	7.47	4.19		0.025	8.43	2.63	0.019
Bacteroidetes	2.63	4.51		0.002	2.85	4.09	0.012
Deinococcus Thermus	–	–		–	1.05	0.51	0.135
Uroviricota	0.91	5.07		<0.001	0.63	4.43	<0.001
Apicomplexa	4.63	2.63		0.560	–	–	–

RA, relative abundance; HC-C, conjunctival swab samples from the healthy control group; DB-C, conjunctival swab samples from the Demodex blepharitis group; HC-M, meibum samples from the healthy control group; DB-M, samples from the Demodex blepharitis group; p-values were compared using Mann–Whitney U test (p<0.05).

**Figure 2:**
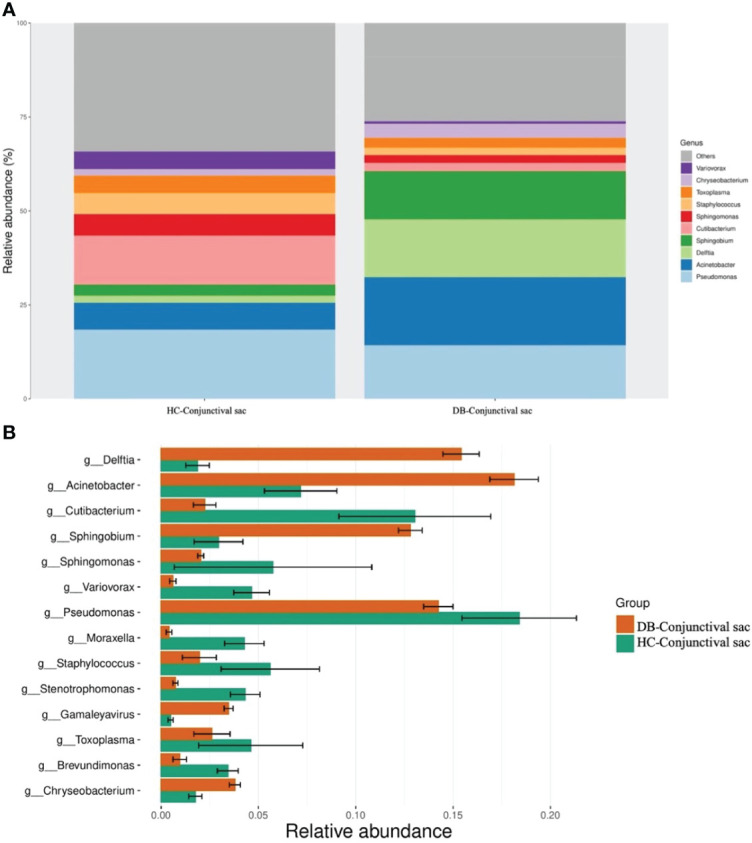
Differences in the relative abundances of microbial genera in the conjunctival sac samples of the control and *Demodex* blepharitis groups. HC, Healthy control; DB, *Demodex* blepharitis.

**Figure 3:**
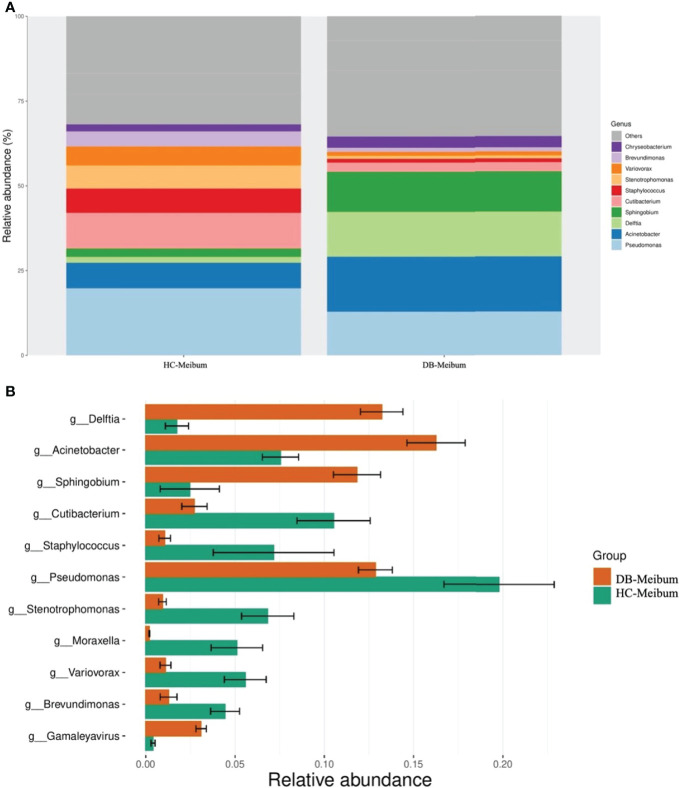
Differences in the relative abundance of microbial genera in the meibum samples of the control and *Demodex* blepharitis groups. HC, Healthy control; DB, *Demodex* blepharitis.

At the genus level, 16 genera in conjunctival swab samples and 18 genera in meibum samples with >1% average RA are shown in **Table 3**. In conjunctival samples, the top-five genera (in the order of RA) of controls and DB groups were *Pseudomonas, Cutibacterium, Acinetobacter, Sphingomonas, Staphylococcus*, and *Acinetobacter, Delftia, Pseudomonas, Sphingobium, Chryseobacterium*, respectively (**Figure 2**; **Table 3**); and the RA of the above genera, except for *Pseudomonas*, was statistically significantly different between the two groups (**Table 3**). Similar to the conjunctival swab samples, the top-five genera of meibum samples (in the order of RA) of controls and DB were *Pseudomonas, Cutibacterium, Acinetobacter, Staphylococcus, Stenotrophomonas*, and *Acinetobacter, Delftia, Pseudomonas, Sphingobium, Chryseobacterium*, respectively (**Figure 3**; **Table 3**); and the RA of these genera was statistically significantly different between the two groups (**Table 3**).

**Table 3:** Genera in the conjunctival swab or meibum samples of controls and *Demodex* blepharitis groups (Relative Abundance>1%).

Samples	Genera	Relative Abundance		*p*-value
		Control	DB	
Conjunctival swab	*Pseudomonas*	18.40	14.24	0.140
	*Cutibacterium*	13.04	2.24	<0.001
	*Acinetobacter*	7.17	18.13	<0.001
	*Sphingomonas*	5.75	2.03	<0.001
	*Staphylococcus*	5.61	1.96	0.003
	*Variovorax*	4.65	0.60	<0.001
	*Toxoplasma*	4.60	2.61	0.580
	*Stenotrophomonas*	4.32	0.74	<0.001
	*Moraxella*	4.27	0.41	0.001
	*Brevundimonas*	3.43	0.96	<0.001
	*Sphingobium*	2.95	12.81	<0.001
	*Delftia*	1.87	15.41	<0.001
	*Chryseobacterium*	1.76	3.79	<0.001
	*Corynebacterium*	0.59	1.47	0.160
	*Gamaleyavirus*	0.49	3.47	<0.001
	*Enterococcus*	0.14	1.02	<0.001
Meibum	*Pseudomonas*	19.78	12.86	0.049
	*Cutibacterium*	10.52	2.72	0.001
	*Acinetobacter*	7.54	16.25	0.002
	*Staphylococcus*	7.16	1.06	0.001
	*Stenotrophomonas*	6.82	0.94	<0.001
	*Variovorax*	5.57	1.11	<0.001
	*Moraxella*	5.10	0.20	0.001
	*Brevundimonas*	4.44	1.28	<0.001
	*Sphingobium*	2.46	11.83	<0.001
	*Chryseobacterium*	2.08	3.29	0.022
	*Delftia*	1.74	13.21	<0.001
	*Ochrobactrum*	1.52	0.04	0.044
	*Comamonas*	1.27	1.04	0.250
	*Orrella*	1.15	0.02	> 0.999
	*Sphingomonas*	0.69	2.23	<0.001
	*Gamaleyavirus*	0.41	3.09	<0.001
	*Paracoccus*	0.17	1.03	0.126
	*Bradyrhizobium*	0.28	1.21	0.016

p-values were compared using Mann–Whitney U test (p<0.05).

**Table 4** shows the species with an average RA>1% in the conjunctival swab or meibum samples of the control and DB groups. In conjunctival swab samples, the top-five species (in the order of RA) of the control and *Demodex* blepharitis groups were *Cutibacterium acnes, Toxoplasma gondii, Moraxella osloensis, Staphylococcus hominis*, *Stenotrophomonas maltophilia*, and *Delftia tsuruhatensis, Acinetobacter johnsonii, Pseudomonas putida, Acinetobacter guillouiae, Acinetobacter* sp. *MYb10*, respectively; and the RA of the above species, except that of *Toxoplasma gondii*, showed statistically significant differences between the two groups. In meibum samples, the top-five species (in the order of RA) in the controls and *Demodex* blepharitis patients were *Cutibacterium acnes, Stenotrophomonas maltophilia, Moraxella osloensis, Staphylococcus hominis, Pseudomonas veronii, and Delftia tsuruhatensis, Sphingobium* sp. *YG1, Acinetobacter johnsonii, Acinetobacter guillouiae, Pseudomonas putida*, respectively; and the RAs of these species were statistically significantly different between the two groups.

**Table 4:** Species in the conjunctival swab or meibum samples of controls and *Demodex* blepharitis groups (Relative Abundance>1%).

Samples	Species	Relative Abundance		*p*-value
		Control	DB	
Conjunctiva swab	*Cutibacterium acnes*	12.92	2.15	<0.001
	*Toxoplasma gondii*	4.60	2.61	0.582
	*Moraxella osloensis*	4.27	0.41	0.001
	*Staphylococcus hominis*	4.14	0.07	<0.001
	*Stenotrophomonas maltophilia*	3.99	0.67	<0.001
	*Sphingomonas* sp. *AAP5*	3.00	0.05	0.043
	*Pseudomonas veronii*	2.97	0.23	<0.001
	*Acinetobacter johnsonii*	2.71	5.84	0.004
	*Pseudomonas fluorescens*	2.39	0.58	0.001
	*Pseudomonas azotoformans*	2.35	0.22	<0.001
	*Pseudomonas stutzeri*	2.27	0.37	0.185
	*Variovorax paradoxus*	1.65	0.20	<0.001
	*Pseudomonas* sp. *TKP*	1.63	0.18	<0.001
	*Delftia tsuruhatensis*	1.57	14.24	<0.001
	*Pseudomonas putida*	1.37	5.54	<0.001
	*Variovorax* sp. *PMC12*	1.28	0.17	<0.001
	*Sphingobium* sp. *YG1*	1.18	11.75	<0.001
	*Pseudomonas extremaustralis*	1.12	0.12	<0.001
	*Sphingomonas melonis*	0.97	1.46	<0.001
	*Staphylococcus epidermidis*	0.54	1.49	0.076
	*Acinetobacter guillouiae*	0.45	5.40	<0.001
	*Acinetobacter* sp. *MYb10*	0.31	3.67	<0.001
	*Chryseobacterium* sp. *JV274*	0.19	1.30	<0.001
Meibum	*Cutibacterium acnes*	10.41	2.69	0.001
	*Stenotrophomonas maltophilia*	6.30	0.84	<0.001
	*Moraxella osloensis*	5.10	0.20	0.001
	*Staphylococcus hominis*	4.84	0.05	<0.001
	*Pseudomonas veronii*	3.60	0.43	<0.001
	*Pseudomonas fluorescens*	2.98	0.67	0.001
	*Pseudomonas azotoformans*	2.77	0.36	<0.001
	*Acinetobacter johnsonii*	2.33	5.34	0.001
	*Pseudomonas* sp. *TKP*	2.09	0.26	<0.001
	*Variovorax paradoxus*	2.07	0.40	<0.001
	*Variovorax* sp. *PMC12*	1.57	0.25	<0.001
	*Ochrobactrum anthropi*	1.47	0.02	0.876
	*Pseudomonas extremaustralis*	1.45	0.19	<0.001
	*Delftia tsuruhatensis*	1.43	12.24	<0.001
	*Brevundimonas naejangsanensis*	1.19	0.22	<0.001
	*Sphingobium hydrophobicum*	1.17	0.67	0.160
	*Orrella dioscoreae*	1.15	0.02	>0.999
	*Pseudomonas putida*	1.11	4.54	<0.001
	*Brevundimonas diminuta*	1.10	0.42	<0.001
	*Staphylococcus epidermidis*	1.08	0.60	0.005
	*Sphingobium* sp. *YG1*	0.94	10.52	<0.001
	*Acinetobacter guillouiae*	0.41	4.57	<0.001
	*Acinetobacter* sp. *MYb10*	0.36	3.34	<0.001
	*Sphingomonas melonis*	0.19	1.21	<0.001
	*Chryseobacterium* sp. *JV274*	0.19	1.11	<0.001

p-values were compared using Mann–Whitney U test (p<0.05).

**Figure 4:**
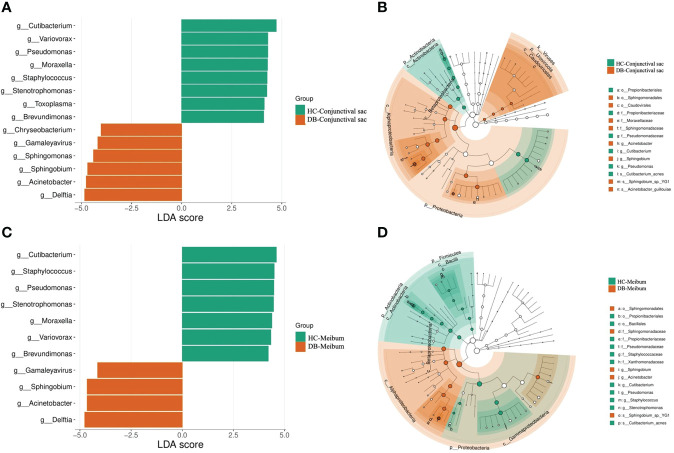
Bacterial biomarkers identified with the linear discriminant analysis effect size (LEfSe) algorithm. Linear discriminant analysis (LDA) scores with the LEfSe tool for taxa, with LDA scores>4 and p<0.05 shown in the histogram **(A, C)**. Cladogram displaying the relations between taxa at different taxonomic levels **(B, D)**. Each circle represents a hierarchy, followed by phylum, class, order, family, and genus. Different phyla are marked with different colors. The size of the nodes represents the taxon abundance. HC, Healthy control; DB, *Demodex* blepharitis.

**Figure 5:**
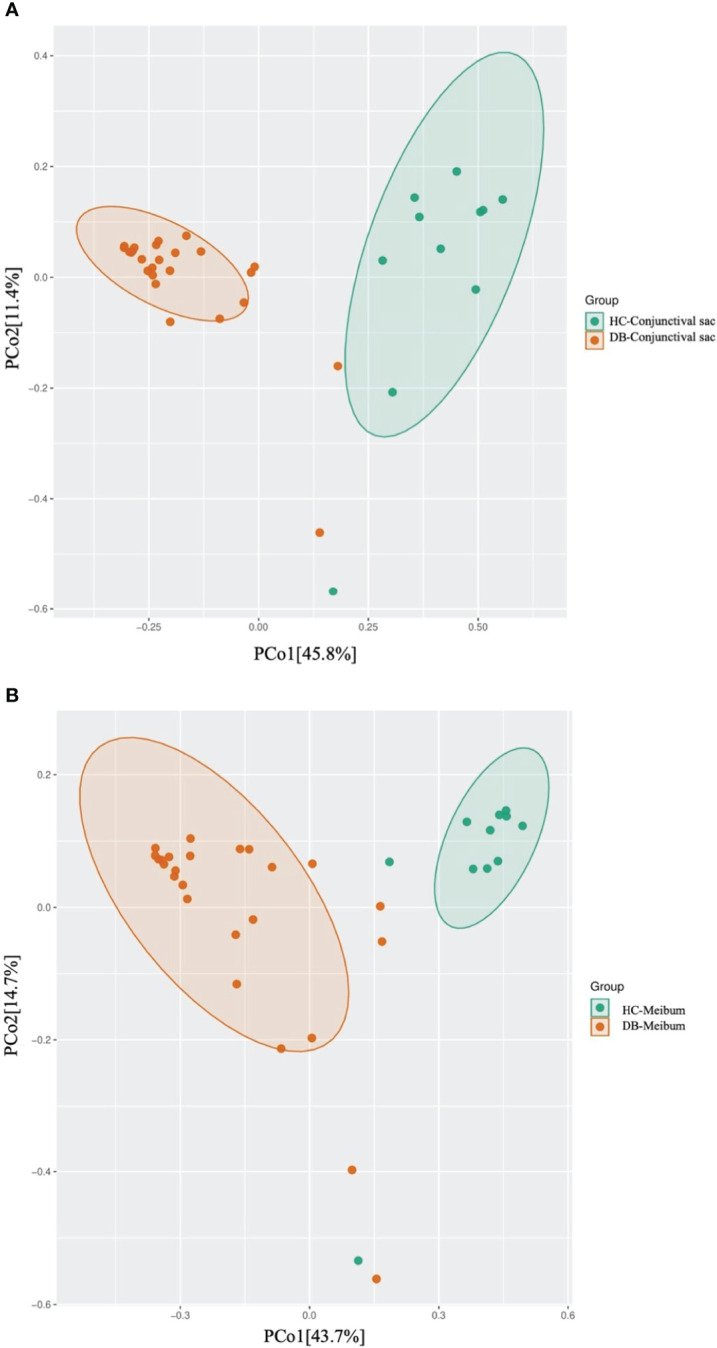
Principal coordinate analysis of two groups of conjunctival sac microbiome **(A)** and meibum microbiome **(B)**. HC, Healthy control; DB, *Demodex* blepharitis.

Linear discriminant analysis (LDA) combined with effect size measurement (LEfSe) analysis of the tax showed that both conjunctival swab and meibum microbiota of DB were enriched in *Sphingobium* sp. *YG1* (species-level)-*Sphingobium* (genus-level)-Proteobacteria (phylum-level) and *Acinetobacter guillouiae* (species-level)-*Acinetobacter* (genus-level)-Proteobacteria (phylum-level), suggesting that these bacteria could be classified as the potential pathogenic bacterial biomarkers for *Demodex* blepharitis. *Cutibacterium acnes* (species-level)-*Cutibacterium* (genus-level)-Actinobacteria (phylum-level), and *Pseudomonas* (genus-level)-Proteobacteria (phylum-level) were more plentiful in the healthy control group’s conjunctival swab and meibum samples than in the DB group (**Figure 4**). Principal coordinate analysis of the discriminative genera separated all control and DB microbiomes into two distinct clusters both in conjunctival swab samples (p = 0.001, PERMANOVA, **Figure 5**) and meibum samples (p = 0.001, PERMANOVA, **Figure 5**).

### Correlation between microbe abundance and ocular surface parameters in the DB group

To investigate the correlation between microbe abundance and the ocular surface parameters in the DB groups, we chose the species (RA >2.0%) to perform GEE analysis, taking age and gender into account. Correlations between the top species and the ocular surface parameters were shown in **Table 5**. It could be considered that higher RAs of species *Sphingobium* sp. *YG1, Acinetobacter guillouiae, and Pseudomonas putida* in both conjunctival swab and meibum samples, and species *Delftia tsuruhatensis* in conjunctival swab samples, were related to a more severe disease with higher scores of SPEED, LAM, Meiboscore and/or lower scores of FBUT, C,FS and meibum. In contrast, higher RAs of species *Acinetobacter johnsonii, Acinetobacter* sp. *MYb10, and Cutibacterium acnes* in both conjunctival swab samples and meibum samples, were related to the mild degree of disease with lower scores of SPEED, LAM, Meiboscore, and/or higher scores of FBUT, meibu,m and CFS. Only *Toxoplasma gondii* had no significant effect on any ocular surface parameters.

**Table 5:** Associations of ocular surface parameters and the species (RA>2.0%) in the conjunctival swab and meibum samples using generalized estimating equations analysis.

Parameters		SPEED		FBUT		CFS		LAM		Meiboscore		Meibum	
		B	*p*	B	*p*	B	*p*	B	*p*	B	*p*	B	*p*
Conjunctival swab	*Delftia tsuruhatensis*	42.690	0.471	-32.677	0.045	10.096	0.443	27.181	0.003	11.418	0.436	-169.029	0.045
	*Sphingobium* sp. *YG1*	-91.930	0.079	3.580	0.801	-31.090	0.008	22.703	0.003	-13.965	0.383	-59.689	0.456
	*Acinetobacter johnsonii*	-88.005	0.069	23.614	0.214	46.552	0.017	8.468	0.221	-42.206	0.008	265.586	0.010
	*Pseudomonas putida*	216.657	0.041	90.436	0.050	1.948	0.946	-47.220	0.035	51.429	0.100	127.588	0.421
	*Acinetobacter guillouiae*	327.494	0.022	-103.627	0.055	-64.503	0.108	-9.500	0.675	84.916	0.029	-185.377	0.492
	*Acinetobacter* sp. *MYb10*	-567.596	0.043	149.927	0.035	73.283	0.230	-105.239	0.002	-114.866	0.011	397.510	0.393
	*Toxoplasma gondii*	-16.200	0.476	-7.552	0.234	8.315	0.179	-0.127	0.972	-4.419	0.386	-20.332	0.562
	*Cutibacterium acnes*	67.625	0.025	2.815	0.811	6.257	0.512	-9.608	0.046	-6.447	0.316	-55.906	0.316
Meibum	*Delftia tsuruhatensis*	-83.092	0.131	-4.454	0.842	-6.232	0.648	2.567	0.816	15.123	0.193	18.837	0.741
	*Sphingobium* sp. *YG1*	54.884	< 0.001	-13.895	0.054	-2.045	0.566	2.875	0.589	7.776	0.026	-45.989	0.007
	*Acinetobacter johnsonii*	-176.508	0.355	184.555	0.021	18.026	0.691	-5.636	0.940	-42.147	0.416	88.044	0.602
	*Acinetobacter guillouiae*	340.425	0.241	-307.911	0.011	31.226	0.687	-38.106	0.729	243.080	0.002	409.791	0.072
	*Pseudomonas putida*	125.097	0.364	-36.323	0.418	-9.534	0.811	34.370	0.249	-1.360	0.964	-290.036	0.037
	*Acinetobacter* sp. *MYb10*	-163.436	0.698	218.983	0.048	-12.085	0.895	-10.073	0.903	-317.721	< 0.001	-260.527	0.402
	*Cutibacterium acnes*	11.432	0.623	7.857	0.334	0.613	0.911	-14.996	0.001	-0.191	0.976	48.914	0.153

### Kyoto Encyclopedia of Genes and Genomes Pathways Analysis

KEGG pathway analysis of both conjunctival swab and meibum samples of DB and control groups revealed enrichment of functions related to infectious diseases, the immune system, and signal transduction.

**Figure 6:**
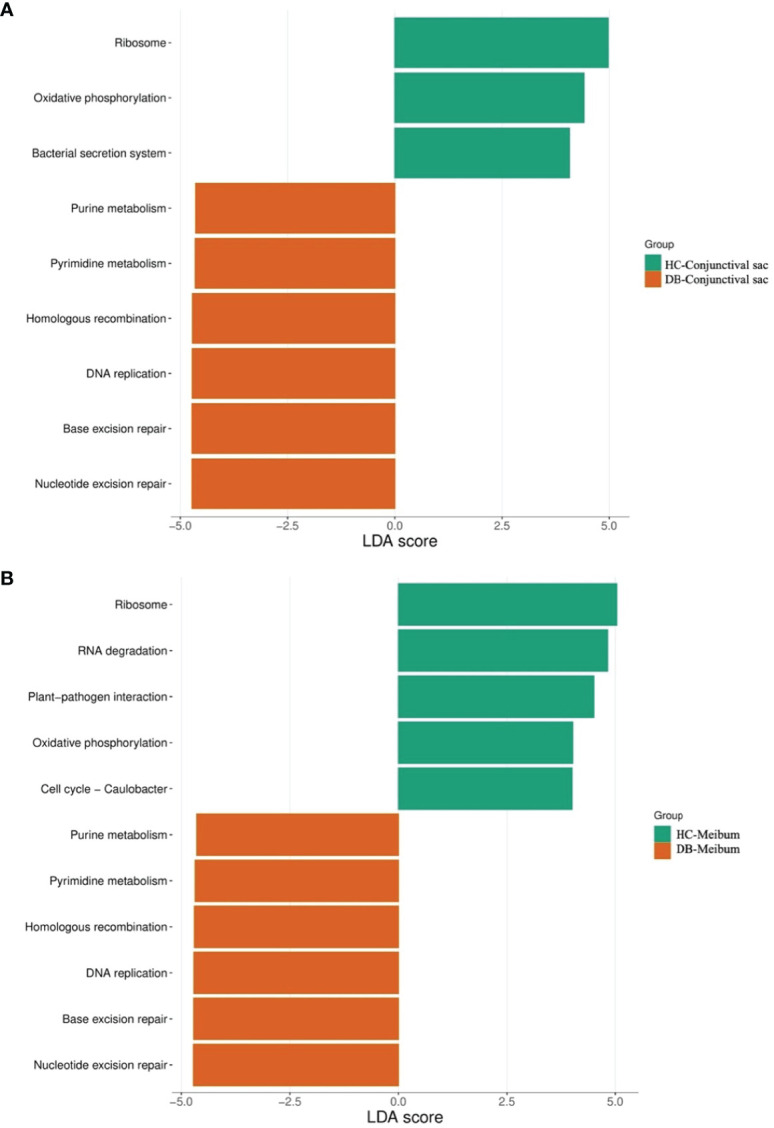
Discriminative Kyoto encyclopedia of genes and Genomes pathways in the conjunctival sac microbiome **(A)** and meibum microbiome **(B)** of the controls and *Demodex* blepharitis groups. HC, Healthy control; DB, *Demodex* blepharitis.

In conjunctival swab samples, LDA with LEfSe showed that nine pathways were significantly different between the control and DB groups (|LDA|>4). Three pathways (ribosome, oxidative phosphorylation, and bacterial secretion system) were more abundant in the control group, and six pathways (purine metabolism, pyrimidine metabolism, homologous recombination, DNA replication, base excision repair, and nucleotide excision repair) were more abundant in the DB group (**Figure 6**).

LDA combined with LEfSe analysis showed eleven pathways that were significantly different between the control and DB groups (|LDA|>4). Five pathways (ribosome, RNA degradation, plant-pathogen interaction, oxidative phosphorylation, cell cycle–caulobacter) were relatively more abundant in the control group, and six pathways (purine metabolism, pyrimidine metabolism, homologous recombination, DNA replication, base excision repair, nucleotide excision repair) were relatively more abundant in the DB group (**Figure 6**).

## Discussion

Blepharitis can arise from inflammation caused by *Demodex* mites on the ocular surface and from secondary bacterial infections (Lee et al., 2010). A comprehensive understanding of the ocular microbiome characteristics associated with *Demodex* mites is essential for understanding the pathogenesis, prevention, and treatment of blepharitis. *Demodex* infests lash follicles and inhabits the sebaceous glands and MGs. The activities of *Demodex* in the MG and their carried pathogens may cause changes in the flora and microenvironment of the conjunctival sac and MG, which causes changes in the meibum composition and aggravates inflammation. At present, there are few studies on the bacterial community of meibum in DB patients. In this study, we used metagenome sequencing to obtain a more comprehensive understanding of the taxonomic and functional complications of the ocular surface microbiome in DB patients.

Alpha-diversity analysis showed that the meibum microbiome diversity (Chao1, ACE, observed, Shannon) in DB and controls was significantly higher than that in the conjunctival swab samples (**Figure 1**). Interestingly, there was no significant difference in the alpha-diversity indices of the meibum microbiome between the two groups, while the conjunctival swab microbiome community population (Chao1, ACE, observed) from DB was significantly lower than that in the controls (p=0.002, 0.001, 0.047, respectively). *Demodex* infestation seems to change mainly the diversity of the microbiome in the conjunctival sac rather than that in the meibum. This may have occurred since we discarded the outer segment of the meibum in the MG and collected the deeper meibum, which might have been minimally affected by *Demodex* mites. This means that even in patients with *Demodex* infection, the microbiome in the meibum did not change significantly as compared with the normal controls since *Demodex* activity was limited to the ocular surface and the proximal opening of the MG, and it did not penetrate the deeper parts of the MG. Moreover, *D. brevis* parasites more obviously influence the MGs, making it more difficult to detect from epilated eyelashes than *D. folliculorum* that reside in eyelashes and follicles. In this study, we also diagnosed DB by detecting mites on the eyelashes; however, the number or absence of *D. brevis* was not clear. Although there was no significant difference in the alpha-diversity of the meibum between the two groups, there were significant differences in the dominant flora and their relative abundances at different taxonomic levels.

Lots of previous studies have shown that Proteobacteria (3.90% – 74%), Actinobacteria (5.00% – 64.8%), Firmicutes (3.90% – 41.71%), and Bacteroidetes(1.73% – 41%) were the most dominant phyla in the ocular surface of healthy subjects, dry eye disease (DED), and MGD (Lu and Liu, 2016; Cavuoto et al., 2019a; Yan et al., 2020; Zhang et al., 2021; Zysset-Burri et al., 2021). In this study, the dominant phyla were consistent with previous studies’ results, and the most abundant phylum was Proteobacteria. The average RA of the phyla Proteobacteria and Actinobacteria was significantly higher and that of the phylum Firmicutes was significantly lower in DB patients than in the controls (**Table 1**). Yan et al.’s study demonstrated different results, which reported a higher RA of Firmicutes in the DB group than the healthy controls, and no significant differences in Proteobacteria and Actinobacteria between two groups (Yan et al., 2020). However, some other studies reported similar results to our study, with a higher RA of the phyla Proteobacteria in MGD or patients with *Demodex* infestation than in healthy controls (Lee et al., 2012; Dong et al., 2019; Liang et al., 2021). Combined with the results of the LEfSe analysis of conjunctival swab and meibum samples, an increased prevalence of the Proteobacteria phylum may be an indicator of an unstable ocular microbial community, as also found in gut microbiota (Shin et al., 2015; Litvak et al., 2017).

Bacterial microbiota assessments of the conjunctival swab and meibum from both healthy controls and *Demodex* blepharitis patients identified several of the most common genera on the ocular surface of patients with *Demodex* infestation, MGD, DED, and healthy controls as previous reported, such as *Pseudomonas* (Ozkan et al., 2017; Wen et al., 2017; Ozkan et al., 2019; Borroni et al., 2019; Li et al., 2019; Andersson et al., 2021), *Cutibacterium* (Ozkan et al., 2017; Wen et al., 2017; Ozkan et al., 2019; Borroni et al., 2019; Dong et al., 2019), *Acinetobacter* (Dong et al., 2011; Ozkan et al., 2017; Li et al., 2019; Andersson et al., 2021), *Sphingomonas* (Ozkan et al., 2017; Dong et al., 2019; Andersson et al., 2021), and *Staphylococcus* (Dong et al., 2011; Ozkan et al., 2017; Wen et al., 2017; Dong et al., 2019). In our study, the RAs of genera *Pseudomonas* (meibum, 19.78% vs. 12.86%) and *Cutibacterium* (conjunctival swabs, 13.04% vs. 2.24%; meibum, 10.52% vs. 2.72%) were significantly higher in controls than in the *Demodex* blepharitis group. Andersson et al. also demonstrated that the RA of the genus *Pseudomonas* was markedly higher in healthy controls than in DED groups (24% versus 6%) (Andersson et al., 2021), and they identified *Pseudomonas* as a bacterial biomarker for healthy controls. In contrast, the RAs of the genera *Acinetobacter*, *Sphingobium*, and *Delftia* in *Demodex* blepharitis patients were significantly higher than that in healthy controls. Lee et al. investigated bacterial 16S rRNA genes of eyelash and tear samples from 7 blepharitis patients with *Demodex* infestation and 4 healthy controls using a pyrosequencing method (Lee et al., 2012); and they reported that an increase of *Staphylococcus, Streptophyta, Corynebacterium*, and *Enhydrobacter*, and a decrease of *Cutibacterium* were observed from blepharitis subjects, in terms of the relative abundances. Liang et al. used 16S rRNA sequencing to analyze the conjunctival swab samples of 14 MGD patients with ocular *Demodex* infestation and 17 healthy people, and reported that *Pseudomonas*, *Acinetobacter*, and *Bacillus* were the top three genera in all subjects, and there were more *Acinetobacter*, *Novosphingobium*, and *Anoxybacillus* in the Demodex infestation subjects and fewer *Novosphingobium*, *Lactobacillus*, and *Candidatus Microthrix* in the healthy control group (Liang et al., 2021). Yan et al. also analyzed bacterial 16S rRNA genes of conjunctival swab samples from 30 *Demodex* blepharitis patients and 14 healthy controls, and demonstrated a significantly higher RA of genus *Lactobacillus* and *Bifidobacterium* in *Demodex* blepharitis patients, while the RAs of genera *Cutibacterium* and *Streptococcus* were not statistically different from normal controls (Yan et al., 2020). A culture-based study found that the total colony counts and the incidences of *Cutibacterium acnes* and *Staphylococcus aureus* from the eyelashes of blepharitis with *Demodex* infestation were significantly higher than that of the controls (Zhu et al., 2018). When comparing the above results, we found that there was no consistent conclusion, which may be due to the relatively small samples, inconsistent diagnostic criteria, inconsistent flora detection methods, and sample types, etc.

This study was the first time to use mNGS to determine the species in conjunctival swab samples and meibum samples, to our best knowledge*. Delftia tsuruhatensis* and *Sphingobium* sp. *YG1* was the most abundant species in the *Demodex* blepharitis samples, while *Cutibacterium acnes* was the most abundant species in the control samples. *Delftia tsuruhatensis* was first reported as the most abundant conjunctival flora in patients with conjunctival lymphoma, which is an emerging opportunistic healthcare-associated pathogen that can affect immunocompromised patients (Ranc et al., 2018), and it may change the conjunctival environment through its ability to degrade and utilize glucose oxidatively (Asao et al., 2019). *Sphingobium* sp. *YG1* is a lignin model dimer-metabolizing bacterium newly isolated from sediment and has never been reported on the ocular surface (Ohta, 2018). However, the genus *Sphingobium* had been reported to be abundant in the MGD meibum (Zhao et al., 2020). Interestingly, the correlation between species abundances and clinical parameters in the DB group showed higher RAs of *Delftia tsuruhatensis*, *Sphingobium* sp. *YG1*, *Acinetobacter guillouiae*, and *Pseudomonas putida* were related to the worse ocular surface condition, while *Cutibacterium acnes, Acinetobacter johnsonii, and Acinetobacter* sp. *MYb10* was related to better ocular surface condition. Combined with the LEfSe results, *Sphingobium* sp. *YG1* and *Acinetobacter guillouiae* could be classified as the potential pathogenic bacterial biomarkers for *Demodex* blepharitis, while *Cutibacterium acnes* could be regarded as a friendlier flora for the healthy ocular surface.

The main difference in KEGG pathway analysis of both conjunctival swab and meibum samples between the groups focused on pathways for energy metabolism, genetic information processing, and environmental information processing. Purine metabolism, pyrimidine metabolism, homologous recombination, DNA replication, base excision repair, and nucleotide excision repair were more common in *Demodex* blepharitis patients than in controls. This suggests that the ocular surface of *Demodex* blepharitis patients might have higher levels of DNA damage and repair and a higher frequency of cellular activity in the host than in controls.

This study had some limitations. One of the limitations is the long duration of this study, and we did not collect sampling environment-negative controls, although we adopted strict environmental sterilization during each sample collection. Second, previous studies had revealed that the microbiota diversity on the ocular surface of healthy participants changes with age (Suzuki et al., 2020; Katzka et al., 2021). Participants’ age ranged from 20 to 60 years in this study, and this age range may have affected the results. Thirdly, this is a single-center research and the enrolled population was likely homogeneous to provide generalized conclusions.

Despite these limitations, this study provides novel insights into the ocular surface microbiota in *Demodex* blepharitis patients. The effect of *Demodex* on the ocular surface microbiome was more significant than that on the meibum microbiome. The increasing of phylum Proteobacteria might be an indicator of an unstable ocular microbial community.

## Data availability statement

The data presented in the study are deposited in the NCBI repository, accession number PRJNA856121. Further inquiries can be directed to the corresponding author.

## Ethics statement

The studies involving human participants were reviewed and approved by Ethical Committee of the Eye Hospital of Wenzhou Medical University. The patients/participants provided their written informed consent to participate in this study.

## Author contributions

QD conceptualized the structure. YF, JW wrote the first draft. DW, TL, XS, LL, MZ, ZZ, and XY made a substantial contribution to the content. All authors approved the final version of the manuscript.

## Funding

This study was supported by the Zhejiang Provincial Medical and Health Science, Technology Program of Health and Family Planning Commission (Grant number: 2019RC220)

## Conflict of interest

The authors declare that the research was conducted in the absence of any commercial or financial relationships that could be construed as a potential conflict of interest.

## Publisher’s note

All claims expressed in this article are solely those of the authors and do not necessarily represent those of their affiliated organizations, or those of the publisher, the editors and the reviewers. Any product that may be evaluated in this article, or claim that may be made by its manufacturer, is not guaranteed or endorsed by the publisher.
